# Persistent Periradicular Lesion Associated With Concurrent Root Fracture and Odontogenic Keratocyst: A Case Report

**DOI:** 10.1002/ccr3.71458

**Published:** 2025-11-09

**Authors:** Mehdi Vatanpour, Fatemeh Rezaei

**Affiliations:** ^1^ Department of Endodontics, Faculty of Dentistry, Tehran Medical Sciences Islamic Azad University Tehran Iran

**Keywords:** cone‐beam computed tomography, differential diagnosis, histopathology, odontogenic cysts, periapical diseases, tooth fractures

## Abstract

Persistent periradicular lesions in root canal‐treated teeth present a diagnostic challenge, as they may result from failed endodontic treatment, root fracture, or non‐endodontic pathoses. Accurate diagnosis is essential, given the significant differences in their management. This report describes a case of a persistent periradicular lesion associated with a root canal‐treated maxillary lateral incisor in a 45‐year‐old male. Despite nonsurgical root canal retreatment, a chronic apical abscess persisted. Cone‐beam computed tomography revealed a well‐defined radiolucency and a suspected root fracture. Exploratory surgery with methylene blue staining confirmed the root fracture. Histopathological examination of the curetted intraosseous lesion revealed a cystic lumen lined by para‐keratinized stratified squamous epithelium with palisaded basal cells and surface corrugation, confirming the diagnosis of an odontogenic keratocyst (OKC). The co‐occurrence of a root fracture and an OKC is a rare finding. This case underscores the non‐specific clinical and radiographic features of such entities that can mimic endodontic disease. A definitive diagnosis and successful treatment planning depend on the correlation of clinical, radiographic, and histopathological findings, highlighting the critical role of biopsy in persistent lesions.

**Summary**

- This report of a rare co‐occurrence of a root fracture and odontogenic keratocyst, mimicking a failed endodontic treatment, underscores the importance of a multidisciplinary diagnostic approach, integrating clinical, radiographic, and histopathological findings to achieve an accurate diagnosis and appropriate treatment.

## 1 Introduction

Endodontic lesions caused by infected root canal systems are the most prevalent radiolucent lesions in the periradicular region of the alveolar bone [1]. Histopathological diagnoses of endodontic lesions include radicular cysts, periapical granulomas, and periapical abscesses, which account for 92% of the pathologies observed in periradicular tissues. Non‐endodontic lesions, with a prevalence of roughly 8%, constitute the other category of periradicular radiolucent lesions [1, 2]. These include odontogenic cysts and tumors, non‐odontogenic intraosseous benign cysts and tumors, fibro‐osseous lesions, and primary or metastatic intraosseous malignancies [3].

A thorough clinical and radiographic examination, along with a comprehensive dental and medical history, is essential to differentiate between endodontic and non‐endodontic lesions [4]. Nevertheless, this approach has limitations in certain situations. For example, non‐endodontic lesions may be misdiagnosed as periradicular lesions of endodontic origin, particularly when they are located in the periradicular area of root canal‐treated teeth. In teeth with pulp necrosis, 1% to 3% of biopsies indicate a non‐endodontic origin, with OKC being the most common diagnosis [2].

Persistent periradicular lesions in root canal–treated teeth may also result from root fractures. Diagnosing root fractures in these teeth is complicated because they can mimic failed root canal treatment. Diagnosis should be based on the patient's dental history, clinical signs and symptoms, and radiographic findings. However, characteristic clinical and radiographic signs of root fractures may be absent in some cases [5, 6].

Although these etiologies may mimic persistent periradicular lesions, they require distinct treatments. This underscores the importance of an accurate diagnosis as the first step toward effective and successful treatment. Persistent periradicular lesions resulting from failed root canal treatment can be addressed through either nonsurgical or surgical root canal retreatment. For non‐endodontic lesions, treatment depends on the specific pathology and can range from no intervention to invasive procedures such as marginal resection [1]. Conversely, the management of root fractures varies from conservative approaches to extraction, based on the extension, direction, and location of the fracture [6].

This report presents a unique case of a persistent periradicular lesion in a root canal‐treated tooth, which was ultimately found to be caused by a concurrent root fracture and OKC. This rare combination mimicked an endodontic lesion and posed a significant diagnostic dilemma.

## 2 Case History/Examination

A 45‐year‐old man was referred to the Endodontic Department at the Faculty of Dentistry, Tehran Medical Sciences Branch, Islamic Azad University. His chief complaint was a purulent discharge from the gingiva adjacent to the left maxillary lateral incisor. The patient's medical history was non‐contributory. The dental history indicated that nonsurgical root canal retreatment had been performed on the tooth two years earlier; however, a follow‐up examination revealed a chronic apical abscess and a persistent periradicular lesion. Suspecting a root fracture, an endodontist referred the patient for further assessment and treatment. The patient also reported undergoing a surgical procedure in the same area two decades prior, though no related radiographic records or histopathological findings were available.

**FIGURE 1:**
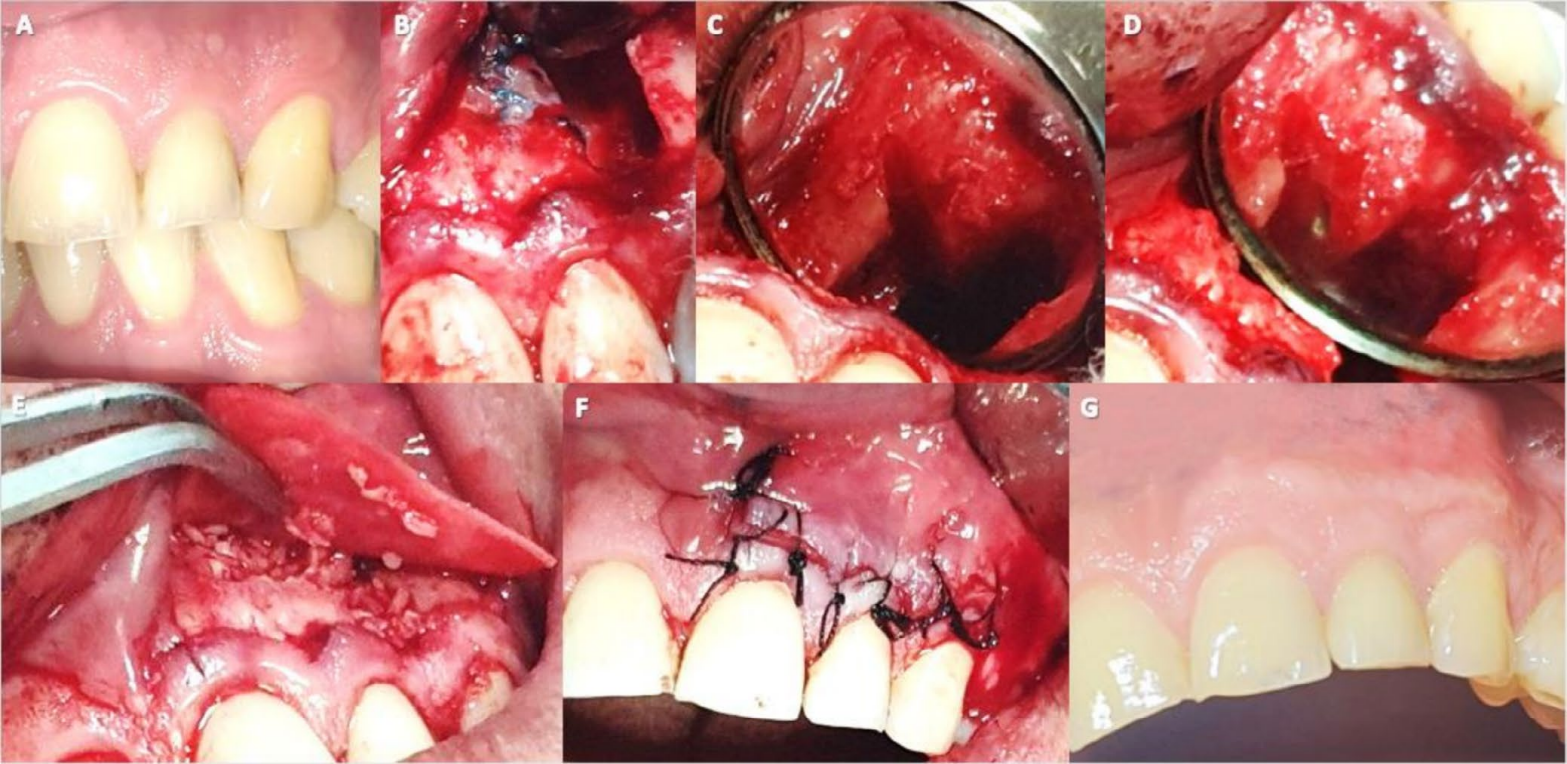
(A) Preoperative photograph indicating the healing sinus tract associated with tooth #10; (B) Staining the root surface with methylene blue confirms the presence of fracture line; (C) The level of resected root at the end of the fracture line; (D) Root‐end preparation filled with CEM cement; (E) Guided tissue regeneration using bone grafts and membrane; (F) Postoperative photograph after suturing; (G) Postoperative photograph after restoration of the crown.

**FIGURE 2:**
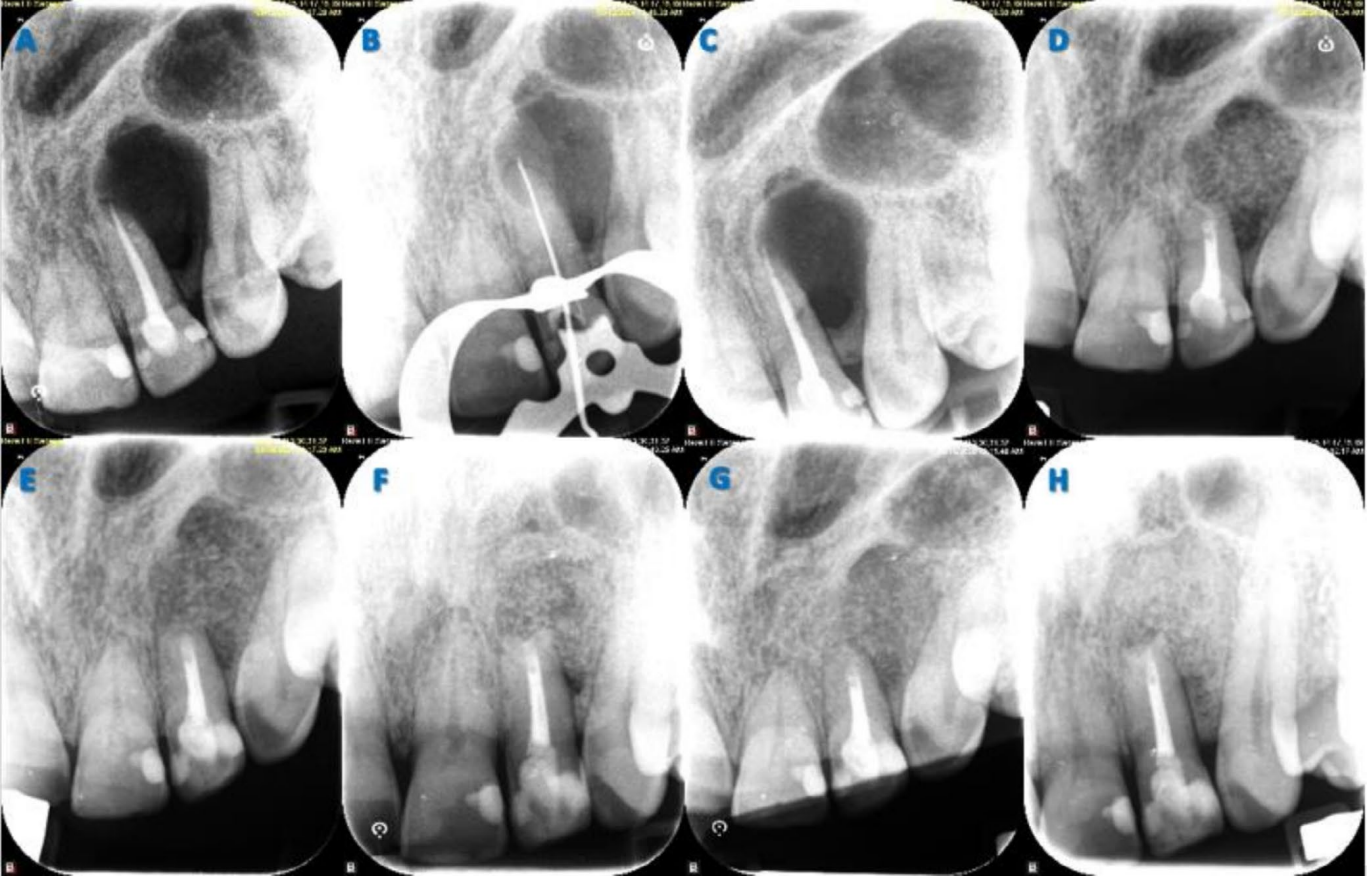
(A) Preoperative periapical radiograph indicating significant periradicular radiolucency; (B) Intraoperative periapical radiograph of length determination; (C) Postoperative periapical radiograph immediately after obturation; (D) Postoperative periapical radiograph immediately after root‐end filling; (E) One‐month follow‐up periapical radiograph; (F) Six‐month follow‐up periapical radiograph; (G) Nine‐month follow‐up periapical radiograph; (H) One year follow‐up periapical radiograph.

**FIGURE 3:**
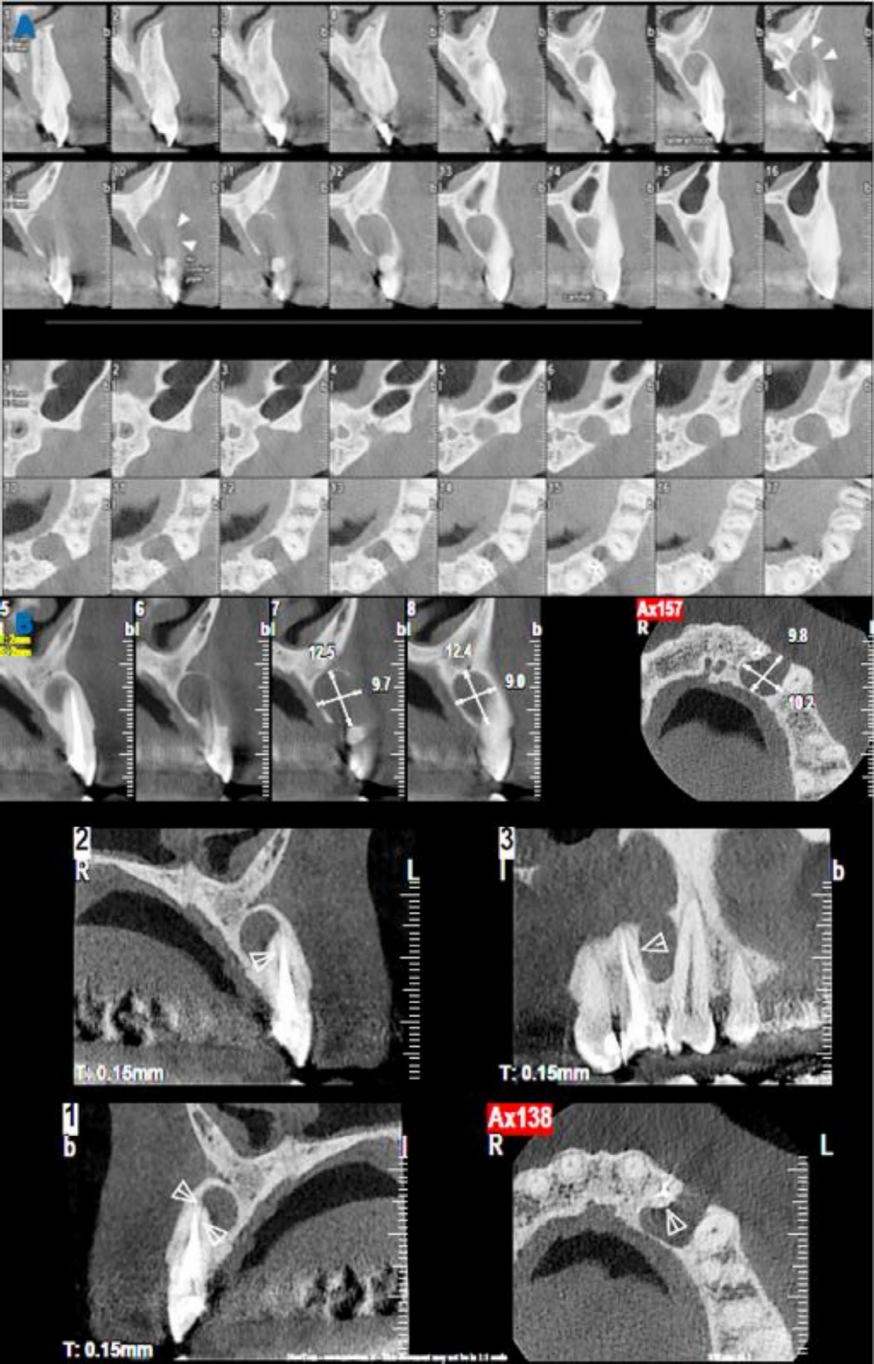
(A) CBCT images (axial and sagittal sections) indicating the extent of the lesion and associated buccal plate perforation; (B) Selected section of the possible fracture line.

Extraoral examination was unremarkable. Intraorally, a healing sinus tract was observed in the attached gingiva of tooth #10 (Figure 1A), alongside evidence of previous restorations. The tooth was non‐responsive to pulp sensibility tests, exhibited no mobility, and was non‐tender to percussion or palpation. Periodontal probing depths were within normal limits. A periapical radiograph revealed evidence of previous root canal treatment, a significant periradicular radiolucency, and recurrent caries (Figure 2A). Cone beam computed tomography (CBCT) images showed a well‐defined radiolucent lesion extending from the distal aspect of tooth #10 to the mesial aspect of the left canine (#11), with associated buccal plate perforation (Figure 3A). Two radiologists independently reviewed the CBCT scans and identified a possible fracture line terminating in the middle third of the root (Figure 3B).

## 3 Differential Diagnosis, Investigations and Treatment

Based on these findings, a diagnosis of chronic apical periodontitis secondary to previous root canal treatment with a potentially infected root canal system and possible root fracture was confirmed. Given the history of prior surgery in the same area, differential diagnoses included lateral radicular cyst, lateral periodontal cyst, and odontogenic keratocyst. A consultation with the restorative department confirmed that crown reconstruction was feasible without intracanal retention, should root resection at the middle third be necessary during exploratory surgery. The patient was presented with treatment options: extraction or exploratory surgery to confirm the presence of a root fracture (and manage it if possible) and to biopsy the intraosseous lesion. The patient chose to preserve the tooth and signed written informed consent for the proposed treatment.

Nonsurgical root canal retreatment was first performed to eliminate potential infection within the root canal system. Local anesthesia was administered via infiltration using 2% lidocaine with 1:80,000 epinephrine (Darupakhsh, Tehran, Iran). The tooth was isolated with a dental dam, and an access cavity was prepared using a small round bur (Jota, Rüthi, Switzerland). Gutta‐percha was removed using Gates Glidden drills (Mani, Tochigi, Japan), stainless steel hand K‐files (Mani, Tochigi, Japan), and NiTi rotary files (V‐Taper Gold, FANTA, Shanghai, China). After determining the working length with Root ZX (J. Morita, Kyoto, Japan) and confirming it radiographically (Figure 2B), cleaning and shaping were carried out. The canal was disinfected using 5.25% sodium hypochlorite (NaOCl; DORADENT, Tehran, Iran), activated with passive ultrasonic activation (Ultra X, Eighteeth, Changzhou, China). It was then irrigated with 17% EDTA (DORADENT, Tehran, Iran) for 1 min, followed by a final 30‐s NaOCl irrigation to remove the smear layer. Any residual NaOCl was flushed with sterile saline, and the canal was dried with paper points (META BIOMED, Cheongju‐si, Republic of Korea). Obturation was completed using the lateral condensation technique with gutta‐percha (META BIOMED, Cheongju‐si, Republic of Korea) and sealer (Adseal; META BIOMED, Cheongju‐si, Republic of Korea). The access cavity was sealed with a temporary restoration (META BIOMED, Cheongju‐si, Republic of Korea) (Figure 2C).

Following retreatment, exploratory surgery was initiated. After preoperative preparation and disinfection of the surgical site with 0.2% chlorhexidine, needle aspiration was performed, yielding only blood. Local anesthesia was administered via infiltration using 2% lidocaine with 1:80,000 epinephrine (Darupakhsh, Tehran, Iran). A full‐thickness mucoperiosteal flap was elevated, extending from teeth #9 to #11. A vertical releasing incision was made at the mesial aspect of tooth #9. Flap reflection revealed a buccal plate perforation. The opening was enlarged with an engine‐driven, saline‐cooled round bur to access the granulation tissue. After irrigating the purulent exudate with saline, the granulation tissue, which exhibited firm epithelial attachment, was carefully excised via curettage. Multiple tissue specimens were collected and stored in 10% formalin for histopathological examination.

The exposed root surface was stained with methylene blue (CERKAMED, Stalowa Wola, Poland) and examined under high magnification using a surgical operating microscope (Zumax, Suzhou, China), which confirmed the presence of a fracture line (Figure 1B). The fractured segment was carefully removed with a bur (Figure 1C). An ultrasonic retro‐tip (NSK, Tokyo, Japan) was used for root‐end preparation, and the cavity was filled with CEM cement (BioniqueDent, Tehran, Iran) (Figure 1D). A postoperative radiograph was taken (Figure 2D). The osteotomy site was grafted with cortical‐cancellous bone powder (150–2000 μm; Kish Tissue Regeneration Corporation, Tehran, Iran) and covered with a resorbable dermal membrane (CenoDerm 0.6–0.9 mm; Kish Tissue Regeneration Corporation, Tehran, Iran) (Figure 1E), ensuring at least 2 mm of membrane extension beyond the osteotomy margins. The flap was repositioned and sutured with 5–0 silk (SUPA, Alborz, Iran) (Figure 1F). Postoperative instructions included ibuprofen 600 mg for pain, amoxicillin 500 mg for antibiotic prophylaxis, and 0.2% chlorhexidine mouthwash twice daily for one week. Sutures were removed after seven days, and the access cavity was permanently restored with composite resin (3M ESPE, St. Paul, MN, USA) one week later (Figure 1G).

## 4 Conclusion and Results (Outcome and Follow‐Up)

**FIGURE 4:**
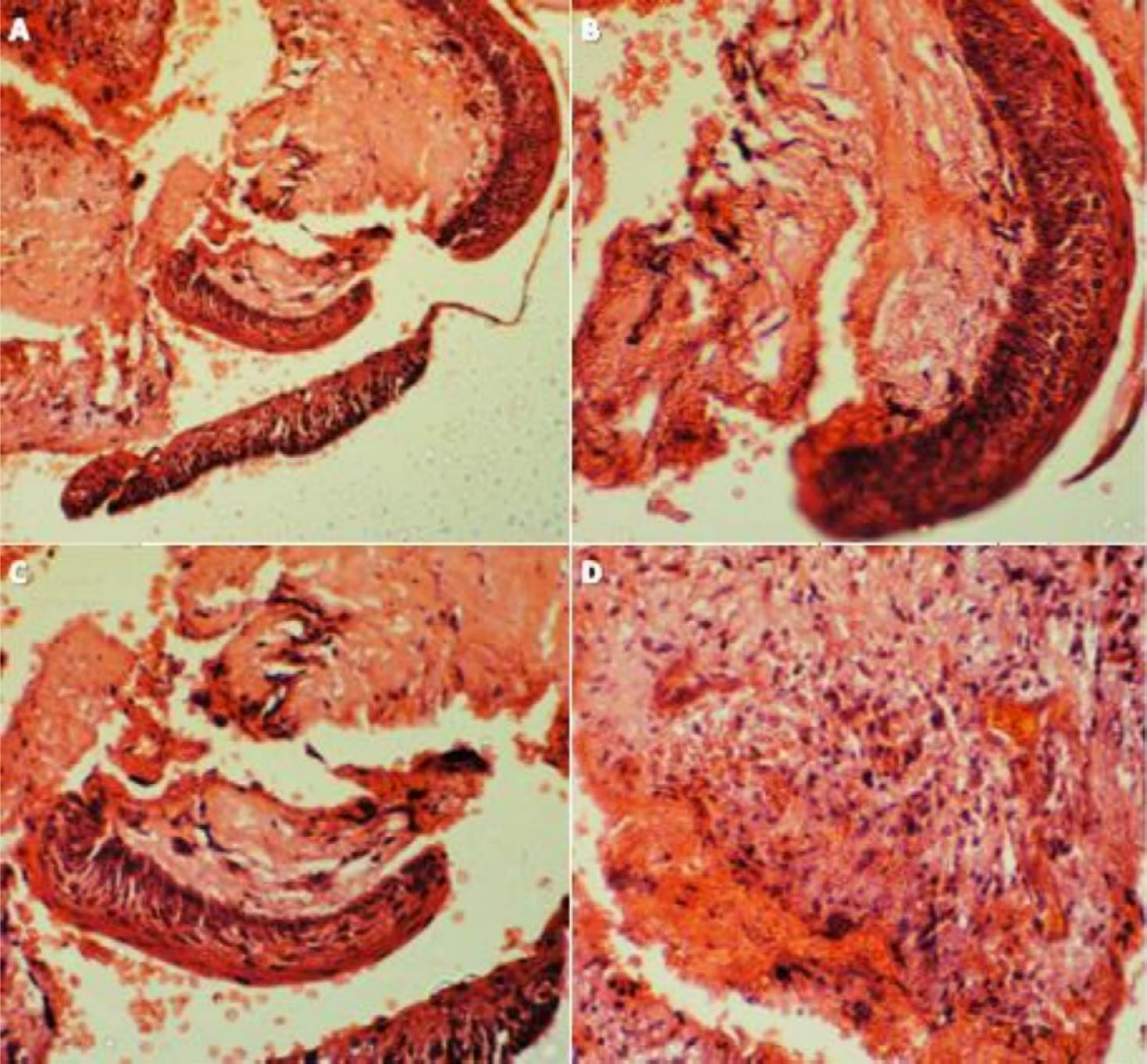
(A–C) A uniform layer of para‐keratinized stratified squamous epithelium, characterized by palisaded basal cells and surface corrugation; (D) The connective tissue with moderate infiltration of lymphocytes.

Histopathological examination revealed a cystic structure partially lined by a uniform layer of parakeratinized stratified squamous epithelium with palisaded basal cells and a surface corrugation (Figure 4A–C). The connective tissue exhibited a moderate lymphocytic infiltrate, scattered bony trabeculae, and areas of hemorrhage, with no signs of malignancy (Figure 4D). The curetted specimen from the intraosseous lesion was definitively diagnosed histopathologically as an odontogenic keratocyst. Due to the high recurrence rate of OKC, the patient was referred to the oral and maxillofacial surgery department. Some surgeons recommended immediate marginal resection, while others favored periodic follow‐up for early recurrence detection. The patient opted for the conservative approach, periodic monitoring (Figure 2E–H).

## 5 Discussion

This report presents a rare case of a concurrent root fracture and odontogenic keratocyst associated with a persistent periradicular lesion in a maxillary left lateral incisor, which posed a significant diagnostic challenge. Endodontic lesions represent the most common pathological finding in periradicular tissues [1]. The presence of a periradicular radiolucent lesion along with a negative response to sensibility testing typically indicates the need for root canal treatment. However, the situation becomes more challenging when such a lesion is associated with a previously root canal‐treated tooth. In the presence of inadequate root canal treatment, the lesion is typically considered a failure of root canal treatment, and nonsurgical root canal retreatment is usually recommended. Surgical retreatment may be indicated in cases where nonsurgical approaches are either ineffective or impossible [7]. However, when the root canal treatment appears adequate, other causes for persistent periradicular lesions, such as root fracture or non‐endodontic lesions, must be considered, as these conditions require fundamentally different treatment approaches.

Root fractures are among the most challenging clinical problems to diagnose and manage. They are defined as fractures involving the dentin, cementum, and root canal system. These fractures are classified according to their orientation (vertical, horizontal, or oblique), location (apical, middle, or coronal third of the root), and extension (incomplete or complete) [5, 8]. Dealing with a root fracture in a root canal–treated tooth can be difficult. In most cases, the fracture remains undiscovered for several years after all treatments have been completed. The diagnosis of root fractures should rely on both radiographic and clinical examinations [9, 10]. However, the definitive diagnosis is often complicated by the absence of distinctive signs and symptoms and characteristic radiographic features. Consequently, differentiating root fractures from other pathologies, such as a failed root canal treatment or periodontal disease, can be challenging [6, 10]. CBCT has shown promise in the early detection due to its superior accuracy compared to traditional radiography [8, 11]. In the present case, a persistent periradicular lesion was observed two years after adequate nonsurgical root canal retreatment. The lesion location correlated with a potential fracture line identified in CBCT by radiologists, suggesting an endodontic origin. Initially, nonsurgical retreatment was performed to address any bacterial infection in the root canal system. However, due to the patient's history of prior surgery in the same area, which raised suspicion of a non‐endodontic lesion, a biopsy was deemed necessary. The patient underwent exploratory surgery to confirm the presence of the fracture and to biopsy the intraosseous lesion.

A tooth with a diagnosed root fracture generally has a poor prognosis, and extraction is often recommended. However, alternative treatment strategies with reported short‐term success exist. Patients should be informed of the benefits and risks of tooth preservation to participate in the choice of their preferred treatment [12]. In selected cases, root resection presents a viable alternative to extraction. This procedure involves removing the fractured portion of the root to preserve the remaining tooth structure. Long‐term success depends on careful case selection to ensure the remaining tooth has adequate periodontal support and can be properly restored [10, 12, 13]. In this case, a root fracture terminating in the middle third of the root was visualized after staining with methylene blue. Given the patient's desire to save the tooth, the presence of sufficient periodontal support, and the restorative department's confirmation that the crown could be restored, the root was resected at the fracture line. Subsequently, root‐end preparation was performed and filled with a calcium silicate‐based cement. Guided tissue regeneration (GTR) is recommended as an adjunct to endodontic surgery to promote bone healing. This technique utilizes bioactive materials such as bone grafts and membranes, alone or in combination. Several studies support the clinical efficacy of GTR in improving outcomes of surgical endodontic procedures, especially for large periapical lesions (≥ 10 mm), regardless of the lesion type [14, 15]. Spontaneous osseous regeneration of a substantial defect does not occur; instead, the space is typically repaired with soft tissue. Regenerative techniques are crucial for recruiting and guiding progenitor cells to differentiate into osteoblasts, cementoblasts, and periodontal ligament cells [16].

In this case, given the uncertain diagnosis before histopathological evaluation and the inability to re‐enter the site postoperatively, bone grafts and membrane were used to promote healing and regeneration of the sizable bone cavity resulting from curettage. A few days later, the histopathologic report revealed the lesion was an odontogenic keratocyst. The OKC is a developmental, non‐inflammatory, benign odontogenic cyst arising from remnants of the dental lamina. It may affect patients of any age or sex; however, it is more often diagnosed in males during their second and third decades. While it can be found in any jaw region, it is frequently observed in the posterior of the mandible, particularly in proximity to the third molar and ramus. OKC is highly aggressive and has a substantial recurrence rate [12, 17, 18]. Radiographic and clinical features of OKC are not specific. This may result in a misdiagnosis, particularly when the lesion is associated with a root canal‐treated tooth. OKC grows within the bone's medullary cavity in an anteroposterior direction without causing apparent bone expansion. It is often asymptomatic and discovered accidentally on radiographs. Symptoms may rarely develop as a result of secondary infection. The radiographic manifestation of OKC might range from diminutive unilocular to extensive multilocular radiolucencies with sclerotic borders [19]. It might look like ameloblastoma, dentigerous cyst, lateral periodontal cyst, or radicular cyst. Definitive diagnosis is histological, characterized by a cystic lumen lined by para‐keratinized, corrugated stratified squamous epithelium with a palisaded basal layer [17, 18].

Although the lesion was curetted as thoroughly as possible, the patient was referred to the oral and maxillofacial surgery department to evaluate the need for further surgery due to the well‐documented high recurrence rate of OKC. The treatment of OKC is a subject of debate, as the literature lacks conclusive evidence to guide optimal treatment selection. Surgical protocols range from conservative to invasive treatments. Enucleation, marsupialization, or its modification, decompression, are considered conservative treatments with a high risk of recurrence due to the thin cystic capsule, which readily detaches and leaves macroscopic remnants or daughter cysts [20, 21]. To improve success rates, enucleation is often combined with adjuvant therapies such as chemical curettage (e.g., Carnoy's solution) or cryotherapy to target residual epithelial cells [21]. Decompression works by creating an opening to relieve intracystic pressure, leading to cyst shrinkage and thickening of the lining for easier subsequent removal. However, it requires significant long‐term patient compliance [20, 21, 22]. More invasive approaches, such as resection, may reduce the risk of recurrence, but they are associated with higher morbidity rates. These approaches are recommended for OKCs that have recurred twice or for those that have undergone malignant changes [17, 22]. According to the patient's preference and following consultation with maxillofacial surgeons, a second surgical procedure was not performed. The patient is currently maintained on a strict recall schedule for follow‐up every three months.

The findings of the present case are supported by consistent clinical, radiographic, intraoperative, and histopathological evidence. Exploratory surgery with staining and high‐magnification inspection confirmed a root fracture, while histopathologic evaluation identified the lesion as an OKC. This correlation between surgical and microscopic findings confirms the diagnosis and underscores the essential role of biopsy in managing persistent periradicular lesions that do not respond to endodontic treatment. Several reports have documented OKCs diagnosed after surgical enucleation of persistent periradicular lesions in root canal–treated teeth [17, 18]. In one case, a second surgery with application of Carnoy's solution was performed to reduce recurrence risk [17], whereas another case was managed conservatively through follow‐up only [18]. Similarly, two reports described non–root canal–treated, non‐vital teeth that did not heal after root canal treatment; subsequent enucleation revealed an OKC. Management again differed regarding the use of Carnoy's solution [22, 23]. Another report documented a root canal‐treated tooth with features suggestive of root fracture and non‐endodontic lesion, which was ultimately diagnosed as a calcifying odontogenic cyst [24]. In contrast, the present case is unique in demonstrating the co‐occurrence of a root fracture and OKC in a previously root canal‐treated tooth. This finding underscores the necessity of a multidisciplinary diagnostic approach, integrating clinical, radiographic, and histopathological evidence to ensure an accurate diagnosis and guide appropriate treatment.

## Author Contributions

**Mehdi Vatanpour:** supervision, writing – review and editing. **Fatemeh Rezaei:** project administration, writing – original draft.

## Consent

Written informed consent was obtained from the patient for the publication of this case report and any accompanying images.

## Conflicts of Interest

The authors declare no conflicts of interest.

## Data Availability

The data that support the findings of this study are available on request from the corresponding author. The data are not publicly available due to privacy or ethical restrictions.
